# Short-term mild hyperventilation on intracranial pressure, cerebral autoregulation, and oxygenation in acute brain injury patients: a prospective observational study

**DOI:** 10.1007/s10877-023-01121-2

**Published:** 2024-02-04

**Authors:** Danilo Cardim, Alberto Giardina, Pietro Ciliberti, Denise Battaglini, Andrea Berardino, Antonio Uccelli, Marek Czosnyka, Luca Roccatagliata, Basil Matta, Nicolo Patroniti, Patricia R. M. Rocco, Chiara Robba

**Affiliations:** 1 Institute for Exercise and Environmental Medicine, Texas Health Presbyterian Hospital, Dallas, TX USA; 2 Department of Neurology, University of Texas Southwestern Medical Center, Dallas, TX, USA; 3 Department of Surgical Sciences and Integrated Diagnostics, University of Genoa, Viale Benedetto XV 16, Genova, Italy; 4 Department of Anesthesia and Intensive Care, IRCCS Ospedale Policlinico San Martino, Genova, Italy; 5 IRCCS Ospedale Policlinico San Martino, Genova, Italy; 6 DINOGMI, University of Genova, Genova, Italy; 7 Brain Physics Laboratory, Division of Neurosurgery, Department of Clinical Neurosciences, University of Cambridge, Cambridge, UK; 8 Department of Neuroradiology, IRCCS Ospedale Policlinico San Martino, Genova, Italy; 9 DISSAL, University of Genova, Genova, Italy; 10 Neurocritical Care Unit, Addenbrooke’s Hospital, Cambridge, UK; 11 Laboratory of Pulmonary Investigation, Carlos Chagas Filho Institute of Biophysics, Federal University of Rio de Janeiro, Rio de Janeiro, Brazil

**Keywords:** Carbon dioxide, Brain injury, Hyperventilation, Cerebral ischemia, Intensive care

## Abstract

Current guidelines suggest a target of partial pressure of carbon dioxide (PaCO_2_) of 32–35 mmHg (mild hypocapnia) as tier 2 for the management of intracranial hypertension. However, the effects of mild hyperventilation on cerebrovascular dynamics are not completely elucidated. The aim of this study is to evaluate the changes of intracranial pressure (ICP), cerebral autoregulation (measured through pressure reactivity index, PRx), and regional cerebral oxygenation (rSO_2_) parameters before and after induction of mild hyperventilation. Single center, observational study including patients with acute brain injury (ABI) admitted to the intensive care unit undergoing multimodal neuromonitoring and requiring titration of PaCO_2_ values to mild hypocapnia as tier 2 for the management of intracranial hypertension. Twenty-five patients were included in this study (40% female), median age 64.7 years (Interquartile Range, IQR = 45.9–73.2). Median Glasgow Coma Scale was 6 (IQR = 3–11). After mild hyperventilation, PaCO_2_ values decreased (from 42 (39–44) to 34 (32–34) mmHg, p < 0.0001), ICP and PRx significantly decreased (from 25.4 (24.1–26.4) to 17.5 (16–21.2) mmHg, p < 0.0001, and from 0.32 (0.1–0.52) to 0.12 (-0.03–0.23), p < 0.0001). rSO_2_ was statistically but not clinically significantly reduced (from 60% (56–64) to 59% (54–61), p < 0.0001), but the arterial component of rSO_2_ (ΔO_2_Hbi, changes in concentration of oxygenated hemoglobin of the total rSO_2_) decreased from 3.83 (3–6.2) μM.cm to 1.6 (0.5–3.1) μM.cm, p = 0.0001. Mild hyperventilation can reduce ICP and improve cerebral autoregulation, with minimal clinical effects on cerebral oxygenation. However, the arterial component of rSO_2_ was importantly reduced. Multimodal neuromonitoring is essential when titrating PaCO_2_ values for ICP management.

**Supplementary Information:**

The online version contains supplementary material available at 10.1007/s10877-023-01121-2.

## Introduction

The scientific literature does not provide a clear consensus regarding the optimal targets of partial arterial pressure of carbon dioxide (PaCO_2_) in patients with acute brain injury (ABI) [1]. Fluctuations in PaCO_2_ can induce rapid and significant alterations in cerebrovascular diameters, subsequently impacting cerebral blood flow (CBF) and cerebral blood volume due to changes in extravascular pH [1]. Hypercapnia, characterized by elevated PaCO_2_ levels, prompts cerebral vasodilation, resulting in an increase in intracranial volume. This effect, particularly pronounced in patients with limited intracranial compensatory reserve, can lead to elevated intracranial pressure (ICP).

The response of vascular tone to changes in PaCO_2_ is more sensitive in the direction of vasodilation compared to vasoconstriction in response to reduced arterial PaCO_2_ levels [2]. PaCO_2_ also has the capacity to influence cerebrovascular autoregulation and reactivity [3], albeit without a well-established impact on clinical outcomes. As a result, modulation of PaCO_2_ is frequently employed to manage and treat intracranial hypertension [4]. However, the utilization of hyperventilation as a strategy in ABI patients has been met with skepticism due to physiological studies suggesting potential risks, such as vasoconstriction-related reduction in CBF and subsequent cerebral ischemia [4]. Conversely, other investigations propose that the controlled implementation of moderate and short-term hypocapnia (target PaCO_2_ of 30–35 mm Hg for 50 min) may be safe, significantly reduce ICP, and have no discernible effects on cerebral metabolism and oxygenation [5, 6].

Given the conflicting evidence surrounding this topic, the most recent recommendations from the European Society of Intensive Care Medicine (ESICM) [1] refrain from offering specific guidance on the therapeutic use of hyperventilation for the management of clinically significant ICP. Hyperventilation is only endorsed in cases of life-threatening conditions, such as brain herniation. However, the Seattle algorithm [7] suggests targeting a PaCO_2_ range of 35–38 mm Hg as Tier 1 therapy, and mild hypocapnia (defined as 32–35 mm Hg) as Tier 2 therapy for managing intracranial hypertension. Short-term hyperventilation has been also advocated as means to reduce raised ICP during plateau waves [8]. It is important to note that no physiological studies have been conducted to assess the effects of this treatment using advanced multimodal neuromonitoring.

In light of these considerations, a prospective observational study was conducted on patients with ABI with the aim to evaluate the changes of ICP, cerebral autoregulation, and regional cerebral oxygenation before and after induction of mild hyperventilation.

## Methods

### Design, ethical approval, and inclusion/exclusion criteria

This single center, prospective observational study was conducted at the General and Neurocritical Care Unit of Policlinico San Martino Hospital, IRCCS for Oncology and Neuroscience, Genova, Italy, a 28-bed level 3 unit which admits critically ill general, trauma and neuro-ICU patients. In our unit, 12 beds are equipped with ICM + software and therefore allow multimodal neurological monitoring. This study was performed according to the “Strengthening the Reporting of Observational Studies in Epidemiology (STROBE)” statement guidelines for observational cohort studies [9] (Additional file 1: ESM Table S1) and was approved by the local ethics review board (Comitato Etico Regione Liguria, protocol n. CER Liguria: 23/2020). According to local rules, written consent was obtained from the patients’ next of kin as all patients were unconscious at the moment of inclusion.

We included adult (> 18 years) patients admitted to our ICU from 1st of March 2021 to 1st of January 2023, following ABI, including traumatic brain injury (TBI), subarachnoid hemorrhage (SAH) or spontaneous intracranial hemorrhage (ICH)), who required intubation and mechanical ventilation. Other inclusion criteria were the need of ICP monitoring, the use of multimodal neuromonitoring of cerebral oxygenation and autoregulation, and who required, according to the Seattle guidelines [7], titration of PaCO_2_ values to mild hypocapnia (32–35 mm Hg) as for tier 2 to control intracranial hypertension. Patients were excluded if they did not undergo multimodal neuromonitoring, had no informed consent signed by the next of kin or, if for clinical reasons, mild hyperventilation was not used to treat ICP.

### Data collection

#### Intensive care management and data collection

Patients were managed according to the most recent Guidelines [10, 11]. Indication for invasive ICP monitoring was set for clinical reasons following the latest Brain Trauma Foundation Guidelines and according to our local policies [10]. Patients’ clinical management of intracranial hypertension was performed according to the Seattle algorithm [7].

Patients were initially sedated with propofol (3–6 mg/kg/h) and/or midazolam (0.03–0.2 mg/kg/h) and fentanyl (0.1–0.8 µg/kg/min) to maintain comfort and avoid agitation. They were kept under ventilator asynchrony using protective tidal volume (6–8 mL per kg of predicted body weight), plateau pressure, fraction of inspired oxygen and respiratory rate were titrated according to respiratory mechanics and to maintain normocapnia (35–45 mm Hg) and SpO_2_ > 94%. Temperature management aimed to avoid fever, and head was elevated at 30–45 degrees optimizing venous return and Hb was maintained > 7 g/dL.

Arterial blood pressure was continuously monitored in the radial or femoral artery zeroed at the level of the right atrium (Baxter Healthcare, CA, United States; Sidcup, UK), aiming at CPP > 60 mm Hg (tier 0 strategies). ICP was monitored continuously using a transducer into the brain parenchymal space or through an external cerebrospinal fluid shunt according to clinical indications.

In case of ICP > 22 mm Hg, tier 1 strategies were applied according to clinical needs and at physicians’ discretion (i.e., CPP 60–70 mm Hg, increased sedation and analgesia, mannitol (0.25-1 g/kg) or hypertonic saline by intermittent bolus). If elevated ICP persisted, a target of mild hypocapnia (hyperventilation) was achieved (32–35 mm Hg), by optimizing tidal volume or respiratory rate.

Data on neuromonitoring were obtained at baseline (T0) with basal PaCO_2_ and after achieving the mild hypocapnia target, allowing 10 min for stabilization (T1). Arterial blood gases were obtained at both time points.

Baseline patients’ demographical data were collected, including age, gender, body mass index, preinjury comorbidities including respiratory, cardiovascular, liver and kidney disease, diabetes mellitus, as well as the type of ABI for ICU admission (i.e., TBI, SAH, ICH). The first available Glasgow coma scale (GCS), as well as pupils’ characteristics (reactivity, iso or anisocoria) and type of ICP monitoring (intraparenchymal or external ventricular drain), were collected. We further recorded the occurrence of main ICU complications including acute distress respiratory syndrome, ventilator-associated pneumonia, acute kidney injury, sepsis, vasospasm, and patients’ clinical outcomes (ICU length of stay, mortality, and neurological status (Glasgow Outcome Score) at ICU discharge).

Computed Tomography (CT) was evaluated by a neuroradiologist and classified according to established neuroradiological scales (Rotterdam, Fisher scales). The most recent available CT scan before inducing mild hyperventilation was compared to the first available CT scan after hyperventilation to assess the occurrence of new ischemic events and worsening of radiological scales.

### Multimodal neuromonitoring

Near infrared spectroscopy (NIRS) through the Root® with O_3_® regional oximetry device (Masimo, CA, United States) was used for the assessment of cerebral oxygenation. This device allows for non-invasive continuous regional cerebral oxygen saturation using bilateral sensors applied in the frontotemporal region. The following parameters were collected from NIRS monitoring: a) rSO_2_, which represents the regional cerebral oxygen saturation, and is derived as the ratio of the concentration of oxyhemoglobin (O_2_Hb) and total hemoglobin (cHb = O_2_Hb + HHb, where HHb is deoxyhemoglobin); b) ΔO_2_Hbi, which is an index associated with changes of concentration of oxyhemoglobin, thus representing predominantly changes in the arterial component of regional oxygen saturation; c) ΔHHbi, an index reflecting changes in concentration of deoxyhemoglobin, approximately representing changes in the venous component of the oxygen saturation; d) ΔcHbi, an index representing the sum of ΔO_2_Hbi and ΔHHbi components (total hemoglobin content) [12, 13].

Continuous monitoring of ICP, ABP and NIRS parameters were collected simultaneously and analyzed using ICM + software (Cambridge Enterprise, Cambridge, UK), which provides real-time analysis of multimodal monitoring modalities at the patient's bedside. Data collected with ICM + were sampled at 100 Hz. NIRS parameters data are output at a 1 Hz rate. Dynamic cerebral autoregulation was assessed using the pressure reactivity index (PRx), which is calculated as the Pearson correlation of 30 consecutive 10-s average values of ABP and ICP over a 5-min moving window [14]. Preserved autoregulation was defined as values of PRx below 0, whereas values > 0.3 were defined as altered CA, as previously described [15, 16]. Static cerebral autoregulation was evaluated through the regression analysis of changes in ABP and ICP, rSO_2_ and ΔO_2_Hbi from T0 to T1.

### Statistical analysis

The Shapiro–Wilk test was used to test the normality of the distribution of the variables. Continuous variables are reported as median and interquartile range (IQR = 25th –75th percentiles). Comparisons between different variables at T0 and T1 were made using repeated measures (paired) t-test for normally distributed variables, while non-normally distributed variables were compared using paired Wilcoxon signed-rank test. Graphical representations of these comparisons are presented as boxplots. Dependent variables were expressed as a change from baseline (T0) in absolute terms (Δ change = T1-T0). The correlations coefficients between systemic and the different neuromonitoring variables were verified using Pearson’s or Spearman’s method, for parametric and nonparametric variables, respectively. All statistical analyses were performed using RStudio software (R version 4.3.1). A p-value < 0.05 was considered statistically significant.

## Results

During the study period, a total of 110 patients were considered for inclusion. Fifty-two patients were excluded as they did not undergo multimodal neuromonitoring and 33 patients were not allocated to a specific bed with ICM + software. A final number of 25 patients were included in the analysis. The characteristics of the patients are presented in Supplementary Material, Table S1. 60% were male, and the median age was 64.7 years (45.9–73.2). Thirteen patients (52%) were admitted for TBI, 7 (28%) for SAH, and 5 (20%) patients for ICH. Six patients (24%) had a history of hypertension. At ICU discharge, median GOSE was 3 (1.8–4.0), and 5 patients died (20%).

### Effect of mild hyperventilation on cerebral and systemic factors

**Fig. 1:**
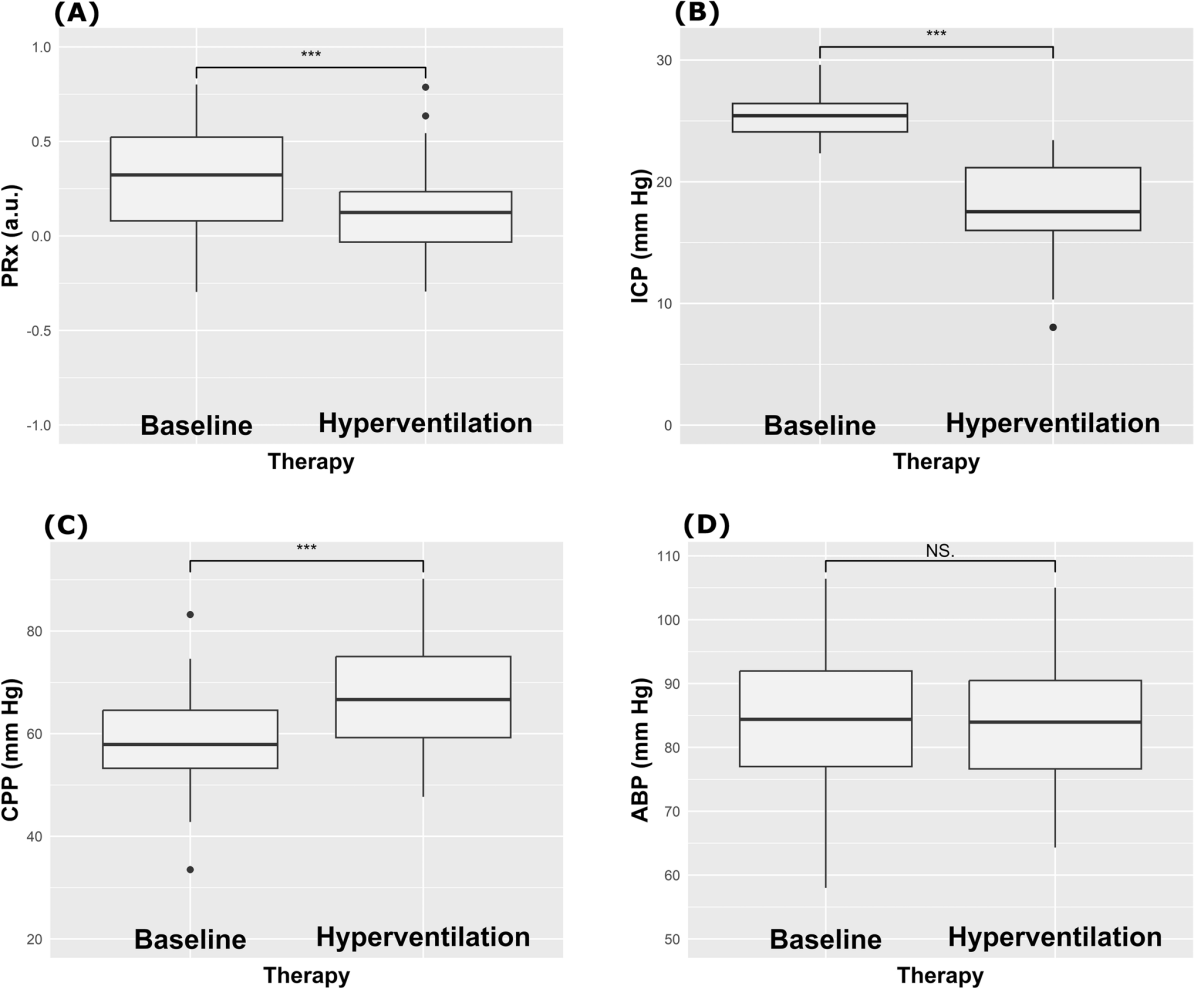
Plots representing the effect of mild hyperventilation on cerebral autoregulation measured with pressure reactivity test (PRx), intracranial pressure (ICP), and cerebral perfusion pressure (CPP) from baseline. NS.: not statistically significant; ***: p < 0.001

**Fig. 2:**
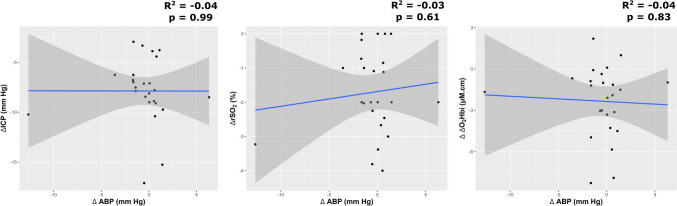
Static cerebral autoregulation plot depicting the relationship between changes in ABP and ICP, rSO_2_ and ΔO_2_Hbi

After mild hyperventilation test, PRx decreased significantly, showing direction toward better cerebrovascular reactivity, from 0.32 (0.1–0.52) to 0.12 (-0.03–0.23), p < 0.0001, Table 1, Fig. 1). Static autoregulation did not indicate any significant relationship between changes in ABP and ICP, rSO_2_ or O_2_Hbi (Fig. 2), suggesting intact cerebral autoregulation during mild hyperventilation.

**Table 1:** Neuromonitoring and systemic parameters at baseline and post-mild hyperventilation test [median (interquartile range)]

	Baseline	Hyperventilation	Δ change	p-value
ICP	25.4 (24.1–26.4)	17.5 (16–21.2)	− 7.8 (− 9 to (− 6.3))	** < 0.0001**
CPP	57.9 (53.3–64.5)	66.7 (59.2–75)	6.6 (4.9–9.4)	** < 0.0001**
ABP	84.4 (77–92)	84 (76.6–90.5)	0 (− 1.4 to 0.6)	0.4
PRx	0.32 (0.1–0.52)	0.12 (− 0.03 to 0.23)	− 0.19 (− 0.26 to (− 0.1))	** < 0.0001**
rSO_2_	60 (56.5–64)	59 (54–61)	− 2 (− 2.5 to (− 0.8))	** < 0.0001**
ΔO_2_Hbi	3.83 (3–6.2)	1.6 (0.5–3.1)	− 1.7 (− 3.3 to (− 0.9))	**0.0001**
ΔHHbi	1.4 (-0.3–3.1)	1 (0.5–2.4)	− 0.17 (− 0.9 to 0.2)	0.9
ΔcHbi	4.6 (2.7–8)	3.5 (1.5–4.4)	− 1.3 (− 5.5 to 1)	**0.008**
SpO_2_	98 (96–100)	97 (96–99)	− 1 (− 3 to 2)	0.36
PaO_2_	96 (92–106)	101 (94–110)	2 (− 2 to 6)	0.11
PaCO_2_	42 (39–44)	34 (32–34)	− 9 (− 10 to (− 6))	** < 0.0001**

*ICP (mm Hg)* intracranial pressure, *CPP (mm Hg)* cerebral perfusion pressure, *ABP (mm Hg)* arterial blood pressure, *PRx (a.u.)* Pressure reactivity index, *rSO*_*2*_* (%)* regional cerebral oxygen saturation, *ΔHHbi (μM.cm)* changes in concentration of deoxygenated hemoglobin (of the total rSO_2_), *ΔO*_*2*_*Hbi (μM.cm)* changes in concentration of oxygenated hemoglobin (of the total rSO_2_), *ΔcHbi (μM.cm)* changes in concentration of total hemoglobin, *SpO*_*2*_* (%)* saturation percent of oxygen, *PaO*_*2*_* (mm Hg)* arterial partial pressure of oxygen, *PaCO*_*2*_* (mm Hg)* arterial partial pressure of carbon dioxide

**Fig. 3:**
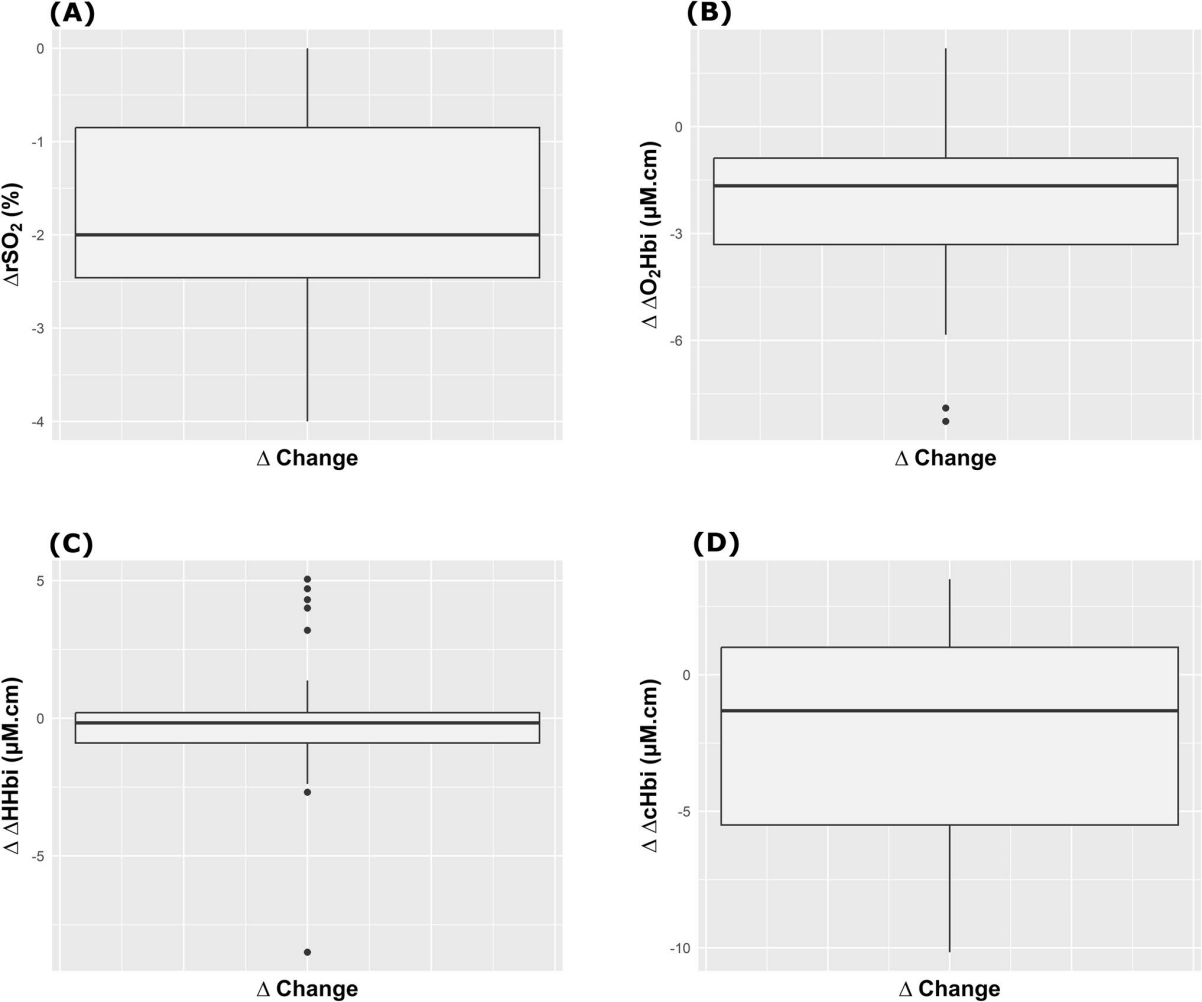
Boxplots representing the effect of mild hyperventilation on absolute changes (∆) in regional cerebral oxygen saturation (rSO_2_), arterial (ΔO_2_Hbi) and venous (ΔHHbi) components of cerebral oxygenation, and concentration of total hemoglobin (ΔcHbi)

ICP significantly decreased (from 25.4 (24.1–26.4) mm Hg to 17.5 (16–21.2) mm Hg, p < 0.0001), as well CPP but not ABP (from 57.9 (53.3–64.5) to 66.7 (59.2–75) mm Hg, p < 0.0001, and from 84.4 (77–92) mm Hg to 84 (76.6–90.5) mm Hg, p = 0.4, respectively) (Table 1, Fig. 1). On the other hand, changes in cerebral oxygenation from T0 to T1 were observed for all parameters except changes in deoxyhemoglobin concentration (ΔHHbi) (Table 2, Fig. 3). rSO_2_ was statistically but not clinically significantly reduced. On the other hand, the arterial component of rSO_2_ (ΔO_2_Hbi, changes in concentration of oxygenated hemoglobin of the total rSO_2_) significantly decreased (Table 1).

**Table 2:** Correlation matrix between changes (Δ) in systemic and neuromonitoring parameters

	ΔICP	ΔCPP	ΔABP	ΔPRx	ΔSpO_2_	ΔrSO_2_	Δ ΔO_2_Hbi	Δ ΔHHbi	Δ ΔcHbi	ΔPaO_2_	ΔPaCO_2_
ΔICP											
r	–	− 0.78*	− 0.31*	− 0.15*	− 0.16*	0.12*	− 0.21*	0.30*	0.18*	0.01*	− 0.01*
p-value	–	** < 0.0001**	0.13	0.47	0.44	0.57	0.32	0.14	0.40	0.98	0.97
ΔCPP											
r	− 0.78*	–	0.67*	0.22	0.16*	0.06	0.07	0.10*	0.01*	− 0.27	− 0.10*
p-value	** < 0.0001**	–	**0.0003**	0.29	0.46	0.77	0.76	0.65	0.96	0.20	0.62
ΔABP											
r	- 0.31*	0.67*	–	− 0.16*	0.13*	− 0.07*	− 0.10*	0.04*	0.09*	0.11*	− 0.06*
p-value	0.13	**0.0003**	–	0.44	0.54	0.75	0.63	0.86	0.68	0.61	0.79
ΔPRx											
R	− 0.15*	0.22	0.16*	–	0.21*	0.15	0.05	− 0.18*	− 0.07*	0.12	− 0.30*
p-value	0.47	0.29	0.44	–	0.31	0.47	0.83	0.39	0.74	0.57	0.15
ΔSpO_2_											
r	− 0.16*	0.16*	0.13*	0.21*	–	0.30*	0.36*	0.10*	0.30*	0.18*	− 0.19*
p-value	0.44	0.46	0.54	0.31	–	0.15	0.08	0.62	0.15	0.39	0.37
ΔrSO_2_											
r	0.12*	0.06	− 0.07*	0.15	0.30*	–	0.21	0.47*	0.51*	0.09	− 0.31*
p-value	0.57	0.77	0.75	0.47	0.15	–	0.32	**0.02**	**0.01**	0.67	0.13
Δ ΔO_2_Hbi											
r	− 0.21	0.07	− 0.10*	0.05	0.36*	0.21	–	0.39*	0.61*	− 0.13	− 0.14
p-value	0.32	0.76	0.63	0.83	0.08	0.32	–	0.06	**0.001**	0.53	0.50
Δ ΔHHbi											
r	0.30*	− 0.10*	0.04*	− 0.18*	0.10*	0.47*	0.39*	–	0.89*	− 0.25*	− 0.26*
p-value	0.14	0.65	0.86	0.39	0.62	**0.02**	0.06	–	** < 0.0001**	0.22	0.21
Δ ΔcHbi											
r	0.18*	0.01*	0.09*	− 0.07*	0.30*	0.51*	0.61*	0.89*	–	− 0.25*	− 0.39*
p-value	0.40	0.96	0.68	0.74	0.15	**0.01**	**0.001**	** < 0.0001**	–	0.22	0.06
ΔPaO_2_											
r	0.01*	− 0.27	− 0.11*	0.12	0.18*	0.09	− 0.13	− 0.25*	− 0.25*	–	− 0.03
p-value	0.98	0.20	0.61	0.57	0.39	0.67	0.53	0.22	0.22	–	0.90
ΔPaCO_2_											
r	− 0.01*	− 0.10*	− 0.06*	− 0.30*	− 0.19*	− 0.31*	− 0.14*	− 0.26*	− 0.39*	− 0.03*	–
p-value	0.97	0.62	0.79	0.15	0.37	0.13	0.50	0.21	0.06	0.90	–

r: correlation coefficient. *Represents Spearman correlation coefficients; the remaining values represent Spearman correlation coefficients

*ICP* intracranial pressure, *CPP* cerebral perfusion pressure, *ABP* arterial blood pressure, *PRx* pressure reactivity index, *rSO*_*2*_ regional cerebral oxygen saturation, *ΔHHbi* changes in concentration of deoxygenated hemoglobin, *ΔO*_*2*_*Hbi* changes in concentration of oxygenated hemoglobin, *ΔcHbi* changes in concentration of total hemoglobin, *SpO*_*2*_ saturation percent of oxygen, *PaO*_*2*_ arterial partial pressure of oxygen, *PaCO*_*2*_ arterial partial pressure of carbon dioxide

PaCO_2_ decreased from 42 (39–44) to 34 (32–34) mm Hg, p < 0.0001, while systemic PaO_2_ did not increase significantly, from 96 (92–106) to 101 (94–110) mm Hg, p = 0.11 (Table 1).

### Correlation between changes in systemic and neuromonitoring parameters

No significant correlations were observed between the changes in ICP and other parameters (Table 2), except with CPP (r = − 0.78, p < 0.0001) (Supplementary Material, Figure S1). Changes in PaCO_2_ were not correlated to systemic or neuromonitoring parameters (Table 2).

## Discussion

In our study, the use of hyperventilation in ABI patients significantly reduced ICP and enhanced dynamic cerebral autoregulation. Post-hyperventilation, regional cerebral oxygen saturation (rSO_2_) experienced a statistically significant reduction, though without substantial clinical implications. Notably, the arterial component of cerebral oxygenation demonstrated a more pronounced reduction, while the venous compartment remained unaffected. These results suggest that the implementation of short-term mild hyperventilation, in line with the current Seattle guidelines [7], can be considered a safe approach for this population. It effectively reduces ICP and enhances cerebral autoregulation with minimal influence on cerebral oxygenation. Other studies also show increasing cerebrospinal compensatory reserve during mild hyperventilation [17]. Our preliminary findings hold significant importance as they contribute to the ongoing discussion on the role and target PaCO_2_ levels in cases of intracranial hypertension and their impact on cerebral hemodynamics.

The determination of optimal PaCO_2_ targets for ABI patients remains inconclusive within the existing literature [1]. While hyperventilation offers potential advantages by reducing cerebrovascular diameter and intracranial volume, thus alleviating ICP, the ensuing hypocapnia and vasoconstriction may lead to reduced cerebral blood flow (CBF) and potential cerebral ischemia [18–20]. Recent recommendations by the ESICM [1] lack consensus on hyperventilation's efficacy in managing elevated ICP, advising its utilization solely as a rescue therapy for brain herniation. The Seattle algorithm proposes a PaCO_2_ titration range of 32–35 mmHg as a Tier 2 strategy for elevated ICP [7]. However, these recommendations rest upon limited evidence, and there is a dearth of physiological studies to assess the effect of this target on cerebral physiology.

A single randomized controlled trial was conducted on this topic three decades ago [21]. The study included control, prophylactic hyperventilation (25 mm Hg), and hyperventilation plus tromethamine groups. However, due to the study's small sample size and methodological constraints, definitive conclusions remain elusive.

Numerous clinical investigations highlight the substantial reduction in CBF and oxygen delivery resulting from hyperventilation [22–24]. An elegant physiological study by Coles et al. [4] employed positron emission tomography (PET) on patients without intracranial hypertension. Decreasing PaCO_2_ from 36 to 29 mm Hg through hyperventilation resulted in a significant decrease in CBF, leading to an escalation of areas at risk of hypoperfusion and ischemia, although local and individual risk thresholds were unknown. Similarly, other authors observed decreased CBF and elevated levels of glutamate, lactate, and lactate/pyruvate ratios following hyperventilation targeting a PaCO_2_ of 24 mm Hg, indicating an altered metabolism [25].

However, other studies propose that mild and short-term hypocapnia may reduce ICP without causing significant pathological changes in brain oxygenation, metabolism, or energy failure [5]. While hyperventilation could potentially reduce global CBF, it may concurrently increase oxygen extraction fraction, leaving cerebral metabolic rate for oxygen unchanged [26]. A recent sub-analysis of the CENTER-TBI study [27], involving 1100 patients and 11,791 PaCO_2_ measurements, revealed a relatively low mean PaCO_2_ of 38.9 (± 5.2) mm Hg, with even lower values applied to patients under ICP monitoring or those with intracranial hypertension. Notably, centers implementing profound hyperventilation (PaCO_2_ < 30 mm Hg) did not exhibit worsened outcomes.

These findings align with our study, which suggests a favorable effect of hyperventilation on ICP without significantly affecting rSO_2_. However, it is crucial to underscore that the arterial component of rSO_2_ exhibited notable impairment, suggesting a pronounced influence on arterial vessel constriction. Despite limited evidence on various NIRS technology rSO_2_ components [13, 28, 29], these results offer a foundation for advanced pathophysiological studies investigating the effect of PaCO_2_ on oxygen delivery and consumption [30]. In this context of titrating PaCO_2_, cerebral oximetry can play an important part in management of ICP, as it can detect changes in the arterial and venous components inside the skull, not just ICP and CPP.

Our study further evaluated the effect of PaCO_2_ on cerebral autoregulation, aiming to enhance our understanding of the intricate interplay between carbon dioxide and perfusion pressure in modulating cerebral circulation, cerebrovascular resistance, and tone. Cerebral autoregulation serves as a compensatory and protective mechanism, ensuring consistent cerebral blood flow despite fluctuations in systemic arterial blood pressure or cerebral perfusion pressure [31]. Impaired autoregulation can lead to secondary brain damage, highlighting the importance of individualizing arterial blood pressure values based on patients' needs and optimal cerebral perfusion pressure [3]. Our findings reveal a substantial reduction in PRx following mild hyperventilation. PRx, signifying the correlation between spontaneous changes in arterial blood pressure and ICP, exhibits negative or reduced values reflecting improved cerebrovascular reactivity [32, 33]. Consequently, mild hypocapnia could enhance dynamic autoregulation (or have minimal effects on it as demonstrated by static autoregulation) by reducing ICP through direct cerebrovascular tone modulation, thereby improving reactivity [3]. Also important, these results shed light on the optimal mechanical ventilation strategies in the neurocritical care patient population, who are often at high risk of respiratory complications due to prolonged ventilation [34, 35], whereby mild hypocapnia can effectively reduce ICP and enhance cerebral autoregulation with minimal influence on cerebral oxygenation.

### Limitations

Several limitations warrant consideration in our study. The modest sample size and single-center, observational design curtail the strength of our conclusions. Heterogeneity within the ABI population fails to specifically address potential PaCO_2_ response differences among subgroups. Prolonged multimodal neuromonitoring could offer more insights into the enduring effects of mild hyperventilation on cerebral dynamics. However, our observational approach aligns with clinical practice protocols, which typically involve short-term hyperventilation. Recognized constraints of NIRS as a surrogate measure of CBF encompass potential extracranial contamination influence, notably in HHb and O_2_Hb signals, the variability of HHb and HbO_2_ reliant on individual-specific scattering coefficients, and the indeterminate impact of venous and arterial factors on measured signals, particularly O_2_Hb, which could introduce variables affecting our study's interpretation. Moreover, it is worth noting that we did not evaluate direct measurements of CBF in the subjects under study.

## Conclusions

This study underscores the benefits of short-term mild hyperventilation, evident through reduced ICP and enhanced cerebral autoregulation in ABI patients. While overall cerebral oxygenation remains intact, the arterial component of rSO_2_ experiences reduction. These findings substantiate the safety and efficacy of mild-short term hypocapnia as a tier 2 therapy for ICP reduction and improved cerebral dynamics in the ABI population. Advanced multimodal neuromonitoring is essential for early complication detection. Larger-scale studies are imperative to corroborate our preliminary outcomes.

### Supplementary Information

Below is the link to the electronic supplementary material.

Supplementary file1 (DOCX 17 kb)

Supplementary file2 (JPG 867 kb)
