# Supplementary material for: Short-term mild hyperventilation on intracranial pressure, cerebral autoregulation, and oxygenation in acute brain injury patients: a prospective observational study
Source: J Clin Monit Comput. 2024 Feb 4;38(4):753–62. doi: 10.1007/s10877-023-01121-2 (PMC11297838; doi:10.1007/s10877-023-01121-2)
Supplement: Supplementary file 1 — Supplementary file1 (DOCX 17 kb) [file 10877_2023_1121_MOESM1_ESM.docx]

| **General characteristics** |  |
| --- | --- |
| Age, years, median [IQR] | 64.7 [45.9; 73.2] |
| BMI, kg/m^2^, median [IQR] | 25.0 [23.4; 26.1] |
| Gender, male/female, n (%) | 15/10 (60/40) |
| **Comorbidities** |  |
| None, n (%) | 6 (24) |
| ≥2 comorbidities, n (%) | 9 (36) |
| Smoke habits, n (%) | 5 (20) |
| Hypertension, n (%) | 6 (24) |
| Diabetes, n (%) | 1 (4) |
| Other endocrine/ metabolic disease, n (%) | 3 (12) |
| Alcohol and/or drugs abuse, n (%) | 1 (4) |
| Kidney injury, n (%) | 1 (4) |
| Liver injury, n (%) | 1 (4) |
| Depression and/or anxiety, n (%) | 1 (4) |
| Cardiovascular disease, n (%) | 1 (4) |
|  |  |
| **Neurological disease and severity** |  |
| Out-of-hospital GCS, points, median [IQR] | 6 [3; 11.5] |
| Anisocoria, n (%) | 8 (32) |
| Type of brain injury |  |
| SAH, n (%) | 7 (28) |
| TBI, n (%) | 13 (52) |
| ICH, n (%) | 5 (20) |
| Type of invasive monitoring |  |
| EVD, n (%) | 9 (36) |
| Intraparenchymal bold, n (%) | 16 (64) |
| TBI, Marshall classification |  |
| I | 0 (0) |
| II | 0 (0) |
| III | 5 (38.4) |
| IV | 4 (30.8) |
| V | 4 (30.8) |
| VI | 0 (0) |
| SAH, Fisher classification |  |
| I | 0 (0) |
| II | 0 (0) |
| III | 0 (0) |
| IV | 7 (100) |
| **ICU outcomes and complications** |  |
| GOSE, points, median [IQR] | 3.0 [1.8; 4.0] |
| ICU complications |  |
| ≥2 complications, n (%) | 8 (32) |
| None, n (%) | 7 (28) |
| Septicemia, n (%) | 13 (52) |
| VAP, n (%) | 8 (32) |
| Acute kidney injury, n (%) | 1 (4) |
| Meningitis, n (%) | 1 (4) |
| Epileptic crisis, n (%) | 2 (8) |
| Hydrocephalus, n (%) | 1 (4) |
| Vasospasm after SAH, n (%) | 1 (4) |
| ICU-LOS, days, median [IQR] | 32.0 [20.0; 52.5] |
| MV duration, days, median [IQR} | 16.0 [9.5; 26.5] |
| Mortality, n (%) | 5.0 (20) |

Table S1. Patient demographics, characteristics, intensive care unit (ICU) complications and patient outcomes.

IQR, Interquartile range; n, number; BMI, body mass index; PBW, predicted body weight; ICU, intensive care unit; TBI, traumatic brain injury; SAH, subarachnoid hemorrhage; ICH, intracranial hemorrhage; GCS, Glasgow Coma Scale; ICP, intracranial pressure; EVD, external ventricular drain; GOSE, Glasgow outcome score extended; ICU-LOS, Intensive care unit length of stay; MV, mechanical ventilation.
