# Supplementary figures and images for: Short-term mild hyperventilation on intracranial pressure, cerebral autoregulation, and oxygenation in acute brain injury patients: a prospective observational study
Source: J Clin Monit Comput. 2024 Feb 4;38(4):753–62. doi: 10.1007/s10877-023-01121-2 (PMC11297838; doi:10.1007/s10877-023-01121-2)

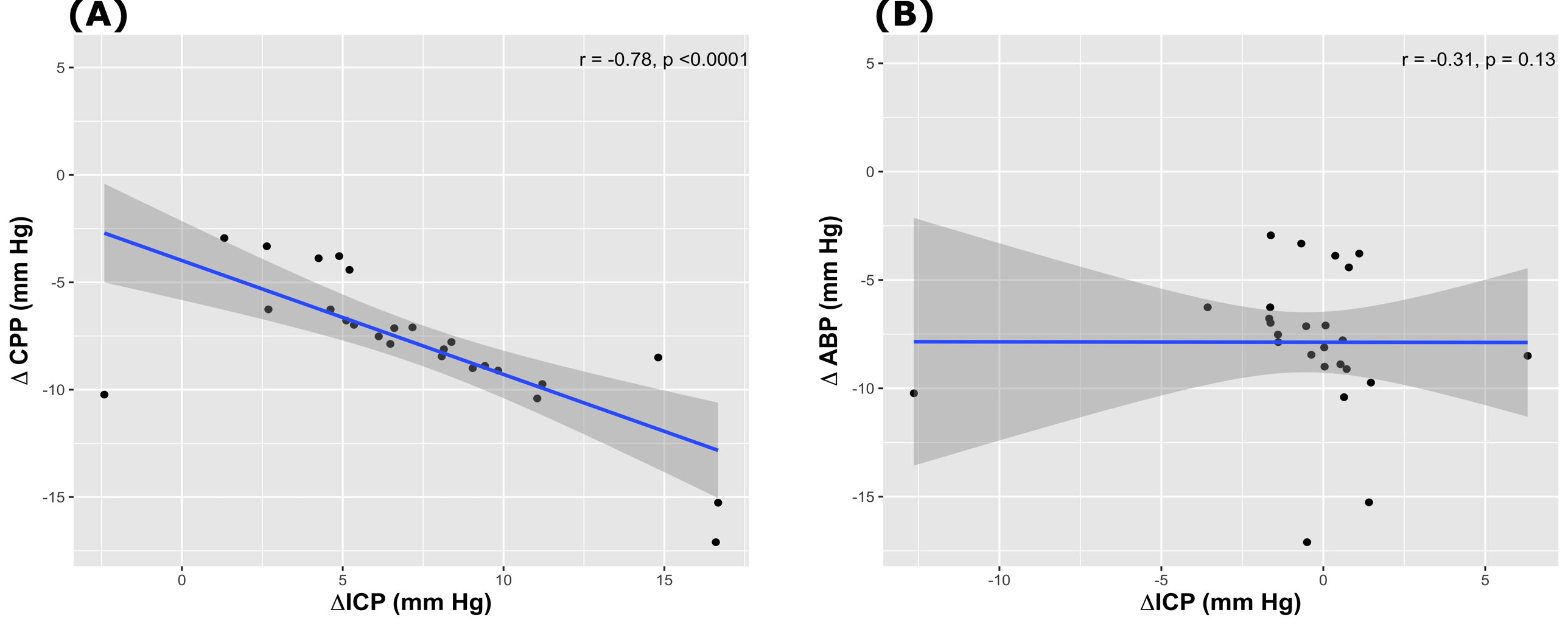

Supplement: Supplementary file 2 — Supplementary file2 (JPG 867 kb) [file 10877_2023_1121_MOESM2_ESM.jpg]
